# Lanthionine, a Novel Uremic Toxin, in the Vascular Calcification of Chronic Kidney Disease: The Role of Proinflammatory Cytokines

**DOI:** 10.3390/ijms22136875

**Published:** 2021-06-26

**Authors:** Alessandra Fortunata Perna, Luigi Russo, Vittoria D’Esposito, Pietro Formisano, Dario Bruzzese, Carmela Vigorito, Annapaola Coppola, Patrizia Lombari, Domenico Russo, Diego Ingrosso

**Affiliations:** 1 First Division of Nephrology, Department of Medical Translational Sciences, University of Campania Luigi Vanvitelli, 80131 Naples, Italy; alessandra.perna@unicampania.it (A.F.P.); carmela.vigorito@unicampania.it (C.V.); patrizia.lombari@unicampania.it (P.L.); 2 Division of Nephrology, Federico II University, 80131 Naples, Italy; luigi.russo@unina.it (L.R.); domenico.russo@unina.it (D.R.); 3 Department of Medical Translational Sciences, Federico II University & CNR/IEOS, 80131 Naples, Italy; vittoria.desposito@unina.it (V.D.); pietro.formisano@unina.it (P.F.); 4 Department of Public Health, Federico II University, 80131 Naples, Italy; dbruzzes@unina.it; 5 Department of Precision Medicine, University of Campania Luigi Vanvitelli, 80138 Naples, Italy; annapaola.coppola@unicampania.it

**Keywords:** vascular calcification, lanthionine, cytokines, hemodialysis, chronic kidney disease

## Abstract

Vascular calcification (VC) is a risk factor for cardiovascular events and mortality in chronic kidney disease (CKD). Several components influence the occurrence of VC, among which inflammation. A novel uremic toxin, lanthionine, was shown to increase intracellular calcium in endothelial cells and may have a role in VC. A group of CKD patients was selected and divided into patients with a glomerular filtration rate (GFR) of <45 mL/min/1.73 m^2^ and ≥45 mL/min/1.73 m^2^. Total Calcium Score (TCS), based on the Agatston score, was assessed as circulating lanthionine and a panel of different cytokines. A hemodialysis patient group was also considered. Lanthionine was elevated in CKD patients, and levels increased significantly in hemodialysis patients with respect to the two CKD groups; in addition, lanthionine increased along with the increase in TCS, starting from one up to three. Interleukin IL-6, IL-8, and Eotaxin were significantly increased in patients with GFR < 45 mL/min/1.73 m^2^ with respect to those with GFR ≥ 45 mL/min/1.73 m^2^. IL-1b, IL-7, IL-8, IL-12, Eotaxin, and VEGF increased in calcified patients with respect to the non-calcified. IL-8 and Eotaxin were elevated both in the low GFR group and in the calcified group. We propose that lanthionine, but also IL-8 and Eotaxin, in particular, are a key feature of VC of CKD, with possible marker significance.

## 1. Introduction

Cardiovascular disease is the most common cause of death in patients with chronic kidney disease (CKD) at all stages of the disease. The high cardiovascular risk may be due in part to excess vascular calcification (VC) [1,2,3,4,5,6,7,8]. The following factors have been associated with medial and/or intimal VC and are disproportionately represented among CKD patients: increasing age and dialysis vintage, hyperphosphatemia and hypercalcemia, oral calcium intake, secondary hyperparathyroidism and adynamic bone disease, and excessive vitamin D administration [9,10,11,12,13,14,15]. Most factors are linked to calcium-phosphate balance; however, it can be argued that VC is present from the initial stages of CKD when mineral metabolism compensation mechanisms are still preserved; therefore, other factors may be at play. In addition, up to now, no specific therapy to prevent progression or to facilitate regression of VC is available, and there is no definite proof that interventions that slow the progression of VC may improve clinically relevant outcomes. Therefore, it is clear that a more detailed understanding of the biochemical processes related to VC is critically needed.

A number of noninvasive methods have been developed for the detection and quantification of VC. Among these, computed tomography (CT) scan detects and quantifies VC, especially coronary artery calcification, even if it does not differentiate between intimal and medial calcium deposition. Several scores, based on plain radiographic imaging or CT scans, are used in clinical studies, among which the Agatston score [16]. The Agatston score quantifies coronary artery calcification (CAC) detected by an unenhanced low-dose cardiac CT scan. The Agatston score allows for early risk stratification for a major adverse cardiac event. Two of us (Russo, D. and Russo, L.) [16] have shown that the extent of CAC was associated with cardiac events in CKD patients with a high calcification score, and VC progression was an independent predictive factor of cardiac events in those starting with a low calcification score. In addition, the presence and extension of VC, irrespective of the arterial site, predict the risk of the all-cause of death in patients starting hemodialysis [5].

Lanthionine is a non-proteinogenic amino acid generated as a side-product of the action of transsulfuration enzymes in hydrogen sulfide (H_2_S) biosynthesis, and it has been demonstrated to be a uremic toxin with several untoward effects [17,18,19,20]; in particular, in the present context, lanthionine, at concentrations comparable to those present in uremic patients, is able to increase intracellular calcium in endothelial cells in culture [21]. It is therefore entirely possible that lanthionine is linked to VC in CKD.

Systemic and local inflammation contributes to VC, but the molecular mechanisms regulating these processes have not been completely defined [22,23]. Infiltrating macrophages release proinflammatory cytokines that drive the influx of lymphocytes and smooth muscle cells. Cellular microvesicles released from macrophages or apoptotic macrophages form a nidus for calcification, and macrophage-derived inflammatory regulators contribute to the disintegration of elastic fibers and matrix components in the vessel wall, all of which may promote VC. It is still not clear if the osteogenic transformation of vascular smooth muscle cells and mineralization are causes or effects of local inflammation. We have shown that bone modifications in uremia are influenced by the capability of the uremic milieu to alter human mesenchymal stem cell osteogenic differentiation [24].

The hypothesis that vascular calcification could be strictly related to both the context of inflammation and calcium derangement, which may in turn be associated with a lanthionine elevation in CKD, is intriguing. Several cytokines, chemokines, and growth factors, which monitor a state of inflammation, are indeed present in circulation, and an extended set of them can be detected and quantitated in a single measurement and relevant data mining system. In this context, we, therefore, assessed the potential role of lanthionine, a sulfur-containing uremic toxin, and a panel of several different cytokines in VC of CKD patients.

## 2. Results

### 2.1. Lanthionine Concentration Is Increased in CKD and Hemodialysis Patients and in Higher VC Grades

**Figure 1:**
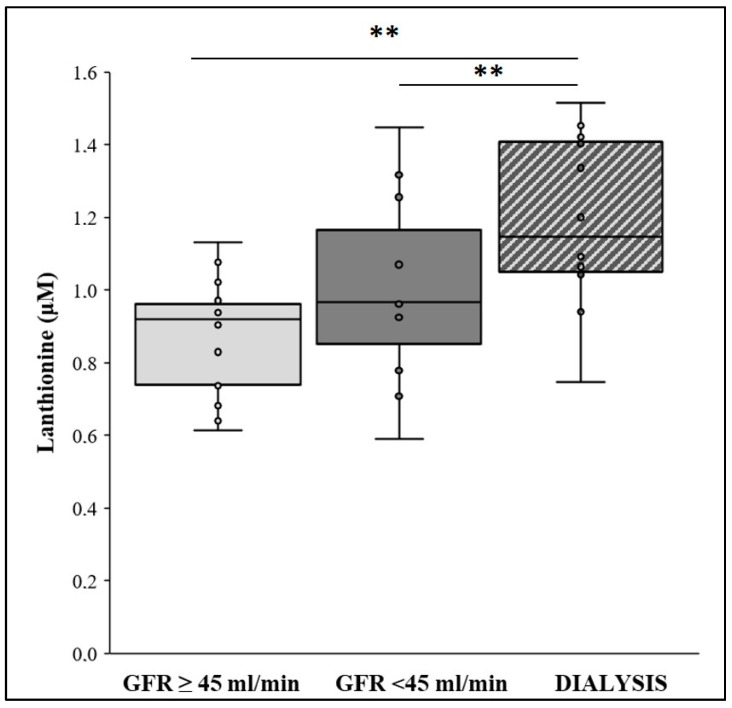
Plasma lanthionine levels in GFR ≥ 45 mL/min/1.73 m^2^, GFR < 45 mL/min/1.73 m^2^, and dialysis patients. Plasma lanthionine was measured by UHPLC/MS system (Ultimate 3000/LTQXL Thermo Scientific), in positive mode, and concentration is expressed as μM values. GFR ≥ 45 mL/min/1.73 m^2^: *n* = 14; 37.83%; GFR < 45 mL/min/1.73 m^2^: *n* = 11; 29.72%; and Dialysis: *n* = 12; 32.43%. Within each box, the horizontal black lines represent the median values. *p*-values: GFR ≥ 45 mL/min/1.73 m^2^ and GFR < 45 mL/min/1.73 m^2^ versus dialysis ** *p* < 0.01, according to the Mann–Whitney U test following the omnibus Kruskal–Wallis test.

Lanthionine concentrations were determined in enrolled patients (*n* = 74) as described in “Methods”. Patients were stratified according to their GFR, <45 mL/min/1.73 m^2^ and ≥45 mL/min/1.73 m^2^; a group of hemodialysis patients (*n* = 12) was also considered. Results showed that there is no significant difference between the two GFR characterized groups (GFR < 45 mL/min/1.73 m^2^ vs. GFR ≥ 45 mL/min/1.73 m^2^; Figure 1), although levels were higher than those reported in the literature for healthy individuals [21]. In addition, in the hemodialysis group, lanthionine was significantly different from the other two groups (Figure 1).

**Figure 2:**
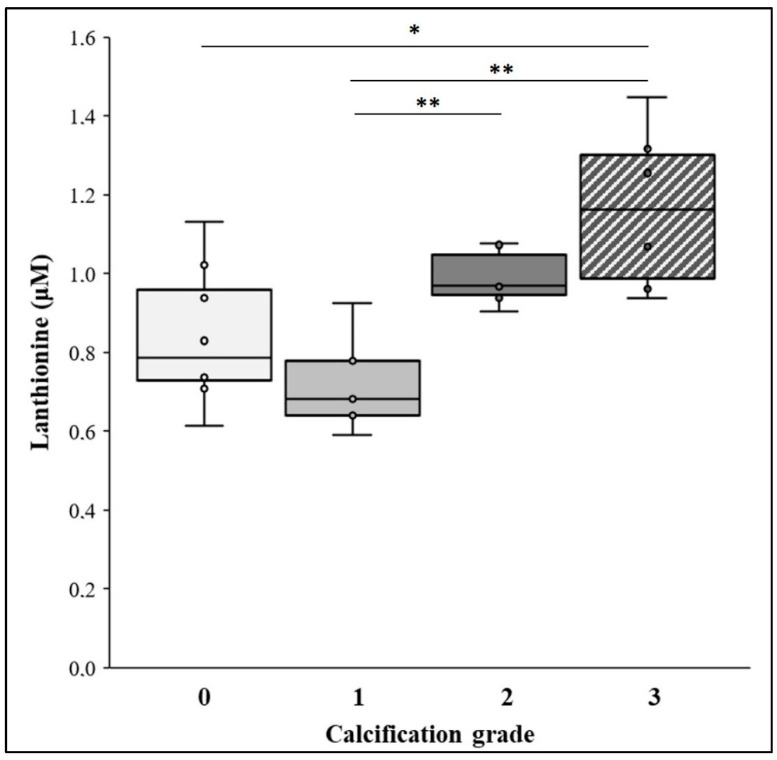
Plasma lanthionine levels and calcification grade. The box whisker plot represents lanthionine (μM) in different calcification grades. The calcification grade was based on the level of coronary artery calcification (CAD), detected by computed tomography (CT) scan, expressed as a calcium score. Agatston score 0 (*n* = 8; 32%), Agatston score 1 (*n* = 5; 20%), Agatston score 2 (*n* = 6; 24%) and Agatston score 3 (*n* = 6; 24%). Within each box, the horizontal black lines represent the median values. *p*-values: Agatston score 0 versus 3 * *p* < 0.05; Agatston score 1 versus 2 and 3 ** *p* ≤ 0.01, according to the Mann–Whitney U test following the omnibus Kruskal–Wallis test.

Patients were also stratified according to the Agatston calcification grading system, as described in “Methods”. The results are depicted in Figure 2. Lanthionine concentrations appeared to be related to the calcification grade. In fact, while there was no difference in serum lanthionine between patient groups displaying 0 and 1 calcification grades, respectively, there was, in patients bearing the highest calcium scores (2 and 3), a significant increase in lanthionine concentrations with respect to groups 0 and 1. This could indicate a relationship between the two indicators, the lanthionine and calcium score, in terms of their respective ability to identify groups of patients with a higher risk of VC.

We computed the Spearman coefficient among the lanthionine amount and all the other cytokines expression levels in CKD patients without finding any significant correlation (see supplemental material, Table S1).

### 2.2. Cytokine Concentrations and GFR Level

**Figure 3:**
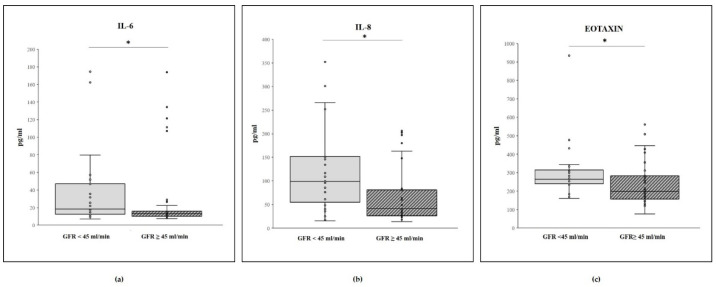
Box plot of plasma level (pg/mL) of (**a**) IL-6, (**b**) IL-8, and (**c**) Eotaxin in GFR <45 mL/min/1.73 m^2^ (*n* = 28, 37.8%) and GFR ≥ 45 mL/min/1.73 m^2^ (*n* = 28, 37.8%) groups. Within each box (25th–75th percentile), the horizontal black lines represent the median values; points outside the whiskers represent outliers, i.e., measurements outside the median ± 1.5 × (75th–25th percentile). *p*-values < 0.05 for all comparisons, according to the Mann–Whitney U test.

Cytokine levels included in an extended array panel were measured in two patient groups according to their GFR values (Table 1). The levels of IL-6, IL-8, and Eotaxin resulted to be significantly increased in the patient group with GFR < 45 mL/min/1.73 m^2^ (Figure 3). At variance, no significant difference between the two groups was observed for the other cytokines.

### 2.3. Cytokine Concentrations and Calcification Score

In Table 2, the levels of the cytokines were compared in patients, divided according to their calcification score into two groups (no calcification = score 0 and calcification = 1 + 2 + 3).

**Figure 4:**
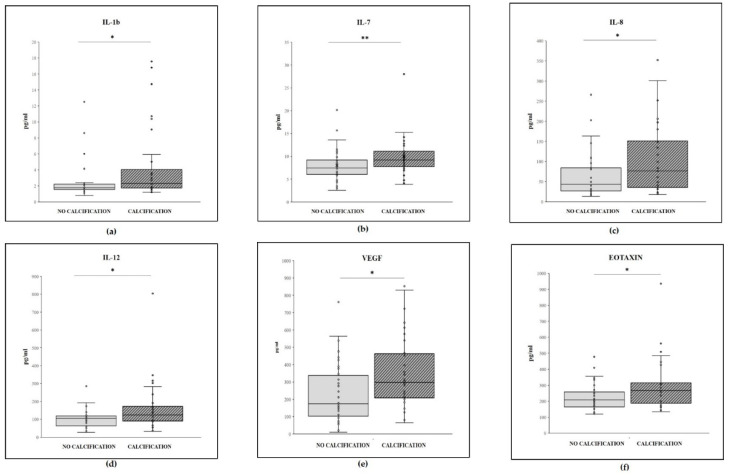
Box plot of plasma level (pg/mL) of (**a**) IL-1b, (**b**) IL-7, (**c**) IL-8, (**d**) IL-12, (**e**) Eotaxin, and (**f**) VEGF (pg/mL) in patients with no calcification (Agatston score 0, *n* = 34; 45.9%) and those with calcification (Agatston 1 + 2 + 3, *n* = 40; 54.1%). Within each box (25th; 75th percentile), the horizontal black lines represent the median values; points outside the whiskers represent outliers, i.e., measurements outside the median ± 1.5 × (75th percentile–25th percentile). *p*-values * <0.05 ** <0.01, according to Mann–Whitney U test.

IL-1b, IL-7, IL-8, IL-12, Eotaxin, and VEGF were significantly increased in the calcified patients with respect to the non-calcified patients (Figure 4). All the other cytokines displayed no significant difference between the two groups.

## 3. Discussion

In this paper, we have analyzed lanthionine, a novel uremic toxin, as well as an extended panel of cytokines, as well as other variables in a population of well-characterized CKD patients with different levels of renal function and on hemodialysis, in order to assess the biochemical bases for the potential role of lanthionine and these inflammatory markers in VC. The conceptual link between lanthionine and cytokines is that both the uremic toxins, thus including lanthionine, and inflammation processes may potentially identify groups of patients with higher cardiovascular risk. Therefore, both markers may identify groups of patients who display more a critical tendency to develop VC.

CKD patients, a population increasing in its number worldwide, have a high cardiovascular risk due to multiple factors, among which we can find the so-called “non-traditional” risk factors such as inflammation and VC. In turn, among the many factors underlining VC and its progression, inflammation is one of the most important [22,23,25].

Lanthionine is a non-proteinogenic amino acid, naturally occurring as a byproduct of H_2_S biosynthesis from cysteine in vivo [17,18,19,20,21]. Lanthionine has been found to be significantly increased in uremic patients [21], and, in these patients, this compound can be considered among the classical uremic retention products responsible for the general clinical features of the uremic syndrome. The first biochemical evidence for this was that lanthionine is able to inhibit H_2_S formation in a hepatocarcinoma cell model (HepG2) [21]. In addition, lanthionine, at concentrations comparable to those actually measured in vivo in patients, is able to induce morphological alterations in zebrafish embryos at early developmental stages and also induced significant alterations of cardiac morphology and function [18]. In endothelial cells, lanthionine hampers H_2_S release reduces both protein content and glutathionylation (a post-biosynthetic regulatory modification) of the transsulfuration enzyme cystathionine-β-synthase [19]. Lanthionine also modifies the expression of miR-200c, miR-423, and VEGF, and, most importantly in this context, it increases intracellular Ca^2+^ levels [19].

We here showed that lanthionine levels increased significantly in hemodialysis patients with respect to the group of patients characterized by their GFR significant variations. This confirms what is already known in the literature; however, in addition, lanthionine increased along with the increase in the TCS. This association is therefore consistent with the findings in in vitro and animal models, such as zebrafish.

IL-6, IL-8, and Eotaxin levels were significantly increased in patients with GFR < 45 mL/min/1.73 m^2^ with respect to those with levels ≥45 mL/min/1.73 m^2^, and IL-1b, IL-7, IL-8, IL-12, VEGF, and Eotaxin increased in calcified patients with respect to the non-calcified. The other markers considered were not significantly different in relation to the GFR or the TCS.

IL-6 is an interleukin that acts as both a pro-inflammatory cytokine and as a myokine involved in the development of obesity-associated insulin resistance [26]. IL-6 is endowed indeed with pleiotropic activities, including an anti-inflammatory action under some conditions [27]. Smooth muscle cells in the tunica media of many blood vessels produce IL-6 as a pro-inflammatory cytokine [28,29]. IL-6 plays a major role in the induction of CRP expression, and it may be involved in the development of many chronic inflammatory diseases with CRP elevation. IL-6 can be elevated in CKD [30].

IL-8 is also known as a *neutrophil chemotactic factor*, and it has two primary functions. It induces chemotaxis in target cells, primarily neutrophils but also other granulocytes, causing them to migrate toward the site of infection. It also stimulates phagocytosis once they have arrived on site. IL-8 is also known to be a potent angiogenesis promoter. In target cells, IL-8 induces a series of physiological responses required for migration and phagocytosis, such as the rise of intracellular Ca^2+^, exocytosis (e.g., histamine release), and the respiratory burst. In addition, IL-8 is involved in cancer progression and metastases [31,32]. Moreover, CKD subjects have IL-8 higher levels than healthy subjects [31]. In a recent study, inorganic phosphate and the known uremic toxin indoxyl sulfate promote VC through interleukin-8 secretion in endothelial cells [33].

Eotaxin-1 (CCL11) is a potent and eosinophil-specific chemoattractant, and it is involved in eliciting inflammation and allergic reactions, as well as asthma. It is released by activated eosinophils and macrophages but also by endothelial and epithelial cells. Furthermore, it is implicated in diseases, such as atherosclerosis, in an independent manner from its action on eosinophils [34,35,36,37], as well as in CKD [38].

IL-1b, IL-7, IL-8, IL-12, Eotaxin, and VEGF increased in calcified patients with respect to the non-calcified.

IL-1b is a cytokine protein with pyrogenic properties, is involved in a variety of cellular activities, including cell proliferation, differentiation, and apoptosis, and is an important mediator of the inflammatory response [39].

IL-7 is a cytokine important for B and T cell development, playing a central role in the homeostasis of the immune system. Its action is required, for example, for successful aging; however, the augmentation of IL-7 above normal levels may disrupt the immune balance [40]. Moreover, while IL-7 deficiency results in lymphopenia, overexpression of IL-7 can cause neoplasia in experimental models [41]. In addition, the stimulation of recombinant IL-7 induces podocyte injury and apoptosis [42]. IL-7 is also hypothesized to be a key regulator in atherosclerosis through its receptor (IL-7r) and through artery tertiary lymphoid organs (ATLOs) [43,44]. IL-7r may contribute to chemokine recruitment in atherosclerosis-associated ATLO development [43,44]. In addition, IL-7/IL-7r can promote the RANKL-mediated osteoclast formation and bone resorption by activating the c-Fos/c-Jun pathway, as well as inducing bone loss in ovariectomized mice, a model of osteoporosis [45]. In our CKD patient population, IL-7 was increased. Up to now, no link with VC has yet been described in the general population or CKD patients, so the present article pinpoints a novel aspect relative to the IL-7 increase.

IL-12 is a cytokine produced by dendritic cells, macrophages, and neutrophils. IL-12 is involved in the differentiation of naive T cells into Th1 cells and enhances the cytotoxic activity of Natural Killer cells and CD8+ cytotoxic T lymphocytes. It also has anti-angiogenic activity. It is also increased in CKD [46,47].

VEGF is a member of the cystine-knot superfamily of extracellular signaling proteins produced by fibroblasts stimulating both vasculogenesis (the de novo formation of the embryonic circulatory system) and angiogenesis (the growth in blood vessels from pre-existing vasculature). It is part of the system that restores the oxygen supply to tissues when blood circulation is inadequate, such as in hypoxic conditions [48]. It is increased in CKD [49]. We have previously shown that lanthionine is able to decrease total VEGF; in particular, 12 h treatment with lanthionine determines a general downregulation of the VEGFA expression, except in the case of isoform 165, and this is consistent with decreased mRNA and protein levels. Longer treatments showed a rebound effect. The increase showed here is therefore likely due to the more pronounced effects of CKD on VEGF with respect to lanthionine.

IL-8 and Eotaxin are the only cytokines increased in both the low GFR and in the calcified patients. Up to now, no link with VC has been yet described in the general population or CKD patients. The results of the present work, indeed, set the biochemical basis to shed new light on the potential pathophysiological aspects of the interactions between cytokines action, as inflammatory mediators and markers, and lanthionine, a uremic toxin that is active on calcium homeostasis in various systems [19,21,24].

A vast amount of evidence is available on the interconnections between inflammation and alterations of calcium homeostasis in CKD [26,27,31,32,33,37,40]. These alterations can be both adequately monitored and are indeed determinant of clinical symptoms and organ dysfunction [50,51]. In particular, Klotho regulates energy metabolism, also exerting anti-oxidative and anti-inflammatory activities. Klotho modulates the calcium and mineral homeostasis and the triad composed by α-Klotho, fibroblast growth factor-23, and its receptor is involved in the pathogenesis of mineral and bone disorder in CKD [10]. Studies also suggest that Klotho is implicated in vascular disease [52] and is endowed with the cardioprotective effect [53]. The role of Klotho in CKD has been previously studied by us as well as and by other groups [10,51,54,55].

In general, relative to the issue of the increase in cytokines/chemokines in CKD, this increase could be a compensatory response to an altered uremic milieu, or they can represent uremic retention products or both. If they are causally linked to VC through various potential mechanisms, this represents an area of an ongoing investigation; however, their measurements can be useful as they can correspond to advantageous biological markers for the identification of VC in CKD patients. Therefore, cytokines IL-1b, IL-7, IL-8, IL-12, Eotaxin, and VEGF are potentially significant predictors of VC and can be useful in clinical practice even for marker-guided decision making. In addition, it can be argued that if the cytokine alterations found are causative with respect to VC, they can even represent prospective candidates for marker-targeted therapy. Anti-cytokine therapy is available, even if not at the last stages of trial testing, and can be envisioned as a potential therapeutic option [56].

Considering lanthionine, the only therapeutic option available up to now is in dialysis patients because, at least in the hemodialyzed group, lanthionine can be efficiently reduced by approximately 50% in a hemodialysis session. Furthermore, the effects of the common therapeutic measures undertaken to slow down disease progression, such as a low protein diet have not been investigated so far.

The evidence supporting the role of lanthionine as a uremic toxin has been reported in previous works [19,21,24], which therefore explained the inverse correlation between GFR and lanthionine. Further, our previous results provided in vitro evidence for the potential role of uremic toxins in the genesis of inflammation. In conclusion, the lanthionine increase appears to be linked not only to the grade of severity of CKD but also to VC; in addition, a set of important cytokines, known elicitors of inflammation, may be the effectors of vascular dysfunction, thus tipping the scales towards calcification. Consequently, our present results set the biochemical basis to support a mechanistic model, in which altered calcium homeostasis and its determinants (also including lanthionine) and inflammation (and its determinants) are related to each other. Our present results are the first evidence, indeed, on the role of lanthionine on vascular calcifications and vascular alterations brought by inflammatory cytokines in vivo in CKD. This notwithstanding, a complete understanding of all these complex interactions certainly requires further studies.

## 4. Materials and Methods

### 4.1. Patients

A population of consecutive asymptomatic CKD patients was selected, provided that the age was >18 y, at stages 2–5 of CKD, not on dialysis, with a 6-month follow-up period before enrollment. Exclusion criteria were heart failure or presence of coronary heart disease, history of myocardial infarction, coronary bypass surgery, angioplasty, stroke, rapidly progressive kidney disease, cardiac arrhythmias (whose presence would have impeded the correct evaluation of CAC due to artifacts). Hypertension was present in 95% of patients, and diabetes in 15%. At enrollment, a thorough clinical evaluation and routine blood tests were performed, in the morning, under fasting conditions. A sample serum or plasma aliquot was stored at −20 °C for further evaluation.

Patients were divided into two groups on the basis of their glomerular filtration rate (GFR), <45 mL/min/1.73 m^2^ or ≥45 mL/min/1.73 m^2^. This cut-off value was chosen because it is recognized that it can distinctly separate patients with a higher or lower risk of mortality [57]. In fact, since the original KDOQI classification was published, stage 3 CKD (a GFR of 30 to 59 mL/min) has been subdivided into GFR stages 3a and 3b to more accurately reflect the different associations between lower GFR and risk for mortality and adverse kidney outcomes [58,59].

A small population of clinically stable hemodialysis patients was also selected (12 patients). All patients were on either hemodialysis or hemodiafiltration, utilizing polysulfone membranes thrice weekly, Kt/V > 1.4, and not affected by systemic diseases such as lupus erythematosus, diabetes mellitus, cancer, or evidence of other systemic diseases antecedent to renal failure. Patients were also hepatitis C virus antibody negative. Previous transplant patients were excluded. Blood tests on hemodialysis patients were performed, in the morning, under fasting conditions, before dialysis treatment. After withdrawal, sample serum or plasma aliquots were stored at −20 °C for further evaluation. Patients were treated with erythropoietin and other drugs commonly utilized in this population for anemia, hypertension, and secondary hyperparathyroidism, following KDOQI guidelines.

### 4.2. Lanthionine Analysis by UHPLC/MS Method

Serum samples were added to 3 volumes of methanol, left at −20 °C for 30′, then centrifuged at 13,000 rpm for 15’ with a 5804R Eppendorf. Supernatants were filtered with a PVDF 0.2 μm syringe filter (GVS, Zola Predosa, BO, Italy) and loaded on UHPLC/MS system (Ultimate 3000/LTQxl Thermoscientific, Thermo Fisher Scientific Inc, Waltham, MA, USA). Sample separation was performed with a Kinetex 5 μm C18 100 A 4.6 × 150 mm (Phenomenex, Torrance, CA, USA). The mobile phase consisted of (A) 0.1% formic acid in mass grade H_2_O (Biochem Chemopharma, Cosne Cours Sur Loire, France) and (B) 0.1% formic acid in mass grade acetonitrile (Romil, Cambridge, UK). The elution proceeded with a linear grade of 2–40% of (B) at 300 μL/min within 40 min with a column temperature of 35 °C. Mass spectrometry analysis was performed by LTQxl Thermoscientific using an ESI source in positive mode, sheath gas was set at 20, and sweep gas was set at 10. Lanthionine parent mass was set at 209 and collision energy at 20. The calibration curve was made with the following lanthionine standards: 0.25–0.50–1.50–3.00 µM; r^2^ = 0.998. All samples were run in duplicate. Serum samples from all 74 CDK patients were analyzed for lanthionine. In 49 samples, lanthionine was below the LOQ (limit of quantification 0.2 µM in serum matrices; Table S2), and the 25 samples in which lanthionine was detected and quantified were used for comparative statistical analysis with GFR and calcification grade (see supplemental material, Table S2).

### 4.3. Cytokinome Analysis

Serum samples were screened for the concentration of the platelet-derived growth factor (PGDF), interleukin-1 beta (IL-1b), interleukin-1 receptor antagonist (IL-1ra), interleukin-4 (IL-4), interleukin-5 (IL-5), interleukin-6 (IL-6), interleukin-7 (IL-7), interleukin-8 (IL-8), interleukin-9 (IL-9), interleukin-10 (IL-10), interleukin-12 (IL-12), interleukin-13 (IL-13), interleukin-17 (IL-17), Eotaxin, basic fibroblast growth factor (FGF), granulocyte-colony stimulating factor (G-CSF), Interferon gamma (IFNG), interferon gamma-induced protein 10 (IP10), monocyte chemoattractant protein-1 (MCP1), macrophage inflammatory protein-1 alpha (MIP1a), macrophage inflammatory protein-1 beta (MIP1b), Regulated upon Activation, Normal T Cell Expressed and Secreted (RANTES), tumor necrosis factor alpha (TNFa), vascular endothelial growth factor (VEGF). The analysis was performed using the Bioplex multiplex Human Cytokine, Chemokine, and Growth factor kit (Bio-Rad, Hercules, CA, USA) according to the manufacturer’s protocol, as previously described [52]. The magnetic bead-based assay was performed on a Bio-Plex^®^ 200 System (Bio-Rad, Hercules, CA, USA). Data are expressed as pg/mL.

### 4.4. Other Analytes

The Glomerular Filtration Rate (GFR) was evaluated through creatinine clearance, utilizing a 24 h urine collection. Creatinine was measured with an enzymatic assay utilizing isotope dilution mass spectrometry standard calibrated method (Roche Diagnostics S.p.A. Monza, Italy). PTH was measured with an immunometric assay in chemiluminescence (Diagnostic Products Corporation, Los Angeles, CA, USA) and C-reactive protein with an immunoturbidimetric high sensitivity assay system (Roche Diagnostics S.p.A. Monza, Italy).

### 4.5. Calcification Assessment

The presence and extent of CAC (Total Calcium Score, TCS, quantified in Agatston Units, AU) were evaluated with a CT scan with no contrast medium administration. Patients were divided into four groups on the basis of their TCS. The first was represented by patients without coronary calcifications; the second, patients with calcifications, Agatston score 1–100; third group, 101–300; fourth group, >301. In Table 3, the clinical and biochemical characteristics of non-calcified and calcified patients are shown.

### 4.6. Statistical Analysis

All analyses were performed using the statistical platform R Core Team (2021; R: a language and environment for statistical computing. R Foundation for Statistical Computing, Vienna, Austria. URL https://www.R-project.org/, accessed on 31 March 2021). Numerical variables were described using either mean ±SD or median with interquartile ranges (25th–75th percentile). Categorical variables were summarized using absolute frequencies and percentages. Accordingly, comparisons between groups were based on the Student’s t-test for independent samples or the Mann—Whitney U test in case of skewed distribution. In the case of more than two groups, ANOVA or Kruskal–Wallis test were used as omnibus tests. Among-group differences with respect to categorical variables were assessed using the Chi-square test. Due to the exploratory setting of this study, no adjustment for multiple comparisons was performed. All tests were two-sided, and statistical significance was set at *p* values less than 0.05.

## Figures and Tables

**Table 1:** Cytokine extended panel and GFR.

Variable (pg/mL)	GFR < 45 mL/min/1.73 m^2^ (*n* = 28; 37.8%)	GFR ≥ 45 mL/min/1.73 m^2^ (*n* = 46; 62.2%)	*p*-Value (Mann–Whitney U Test)
Hu PDGF-bb	7157 [4929; 8710]	7492 [5802; 9657]	0.382
Hu IL-1b	2.3 [1.7; 4.3]	1.8 [1.5; 3.5]	0.206
Hu IL-1ra	164.9 [119.8; 210.3]	148.8 [127.8; 197.3]	0.895
Hu IL-4	18.2 [15.7; 21]	19.7 [18.2; 21.5]	0.092
Hu IL-5	1.9 [1.1; 2.8]	1.9 [1.4; 2.6]	0.972
Hu IL-6	18.4 [12; 48.1]	13.3 [10.1; 20.9]	**0.033**
Hu IL-7	9.1 [5; 11.2]	8.1 [6.9; 10.3]	0.834
Hu IL-8	99.4 [48.8; 153.1]	41.7 [25.9; 83]	**0.010**
Hu IL-9	36.6 [26.4; 46.7]	33.2 [27.2; 44.3]	0.572
Hu IL-10	25.4 [12.4; 39.2]	22.4 [15.1; 30.1]	0.281
Hu IL-12 (P70)	112.7 [89; 184.7]	111.6 [72.4; 137.4]	0.286
Hu IL-13	12.8 [8.9; 16.8]	11.8 [9.3; 14.2]	0.582
Hu IL-17	160.2 [125.4; 185.7]	163.7 [128; 205.6]	0.569
Hu Eotaxin	264.7 [237.5; 327.4]	198.7 [159.1; 286.7]	**0.009**
Hu FGF basic	36.2 [23.8; 42.1]	30.4 [25.4; 36.7]	0.173
Hu G-CSF	74.1 [54.8; 95.7]	73.6 [66.4; 84.3]	0.599
Hu IFN-g	94.3 [64.2; 127.3]	99.1 [82; 122.7]	0.158
Hu IP-10	1186 [863; 1623]	1003 [764; 1302]	0.057
Hu MCP-1 (MCAF)	95.1 [64.7; 150.3]	106.3 [67.7; 162.5]	0.598
Hu MIP-1a	7.2 [4.9; 11.4]	5 [4.1; 7.4]	0.067
Hu MIP-1b	158.6 [104.2; 297.8]	155.4 [101.7; 210.7]	0.613
Hu RANTES	31,075 [16,841; 37,995]	31,100 [24,342; 36,450]	0.828
Hu TNF-α	39.8 [29.2; 55.5]	45.7 [33.6; 68.2]	0.149
Hu VEGF	293.4 [182.2; 453.2]	232.4 [139; 371.3]	0.179

In bold type *p*-value < 0.05. Legend—HU PDGF-BB—Human platelet-derived growth factor-homodimers B; HU IL-1B—Human interleukin-1 beta; HU IL-1RA—Human interleukin-1 receptor antagonist; HU IL-4—Human interleukin-4; HU IL-5—Human interleukin-5; HU IL-6 Human—interleukin-6; HU IL-7—Human interleukin-7; HU IL-8—Human interleukin-8; HU IL-9—Human interleukin-9; HU IL-10—Human interleukin-10; HU IL-12 (P70)—Human interleukin-12 heterodimers 70 kDa; HU IL-13—Human interleukin-13; HU IL-17—Human interleukin-17; HU EOTAXIN—Human Eotaxin; HU FGF BASIC—Human basic fibroblast growth factor; HU G-CSF—Human granulocyte colony-stimulating factor; HU IFN-G—Human interferon gamma; HU IP-10—Interferon gamma-induced protein 10; HU MCP-1(MCAF)—Monocyte chemoattractant protein-1/monocyte chemotactic and activating factor; HU MIP-1A—Macrophage inflammatory protein-1 alpha; HU MIP-1B—Macrophage inflammatory protein-1 beta; HU RANTES—Human RANTES protein; HU TNF-A—Human tumor necrosis factor alpha; HU VEGF—Human vascular endothelial growth factor.

**Table 2:** Cytokines extended panel and calcification score.

Variable (pg/mL)	No Calcification (*n* = 34; 45.9%)	Calcification (*n* = 40; 54.1%)	*p*-Value (Mann–Whitney U Test)
Hu PDGF-bb	7530 [5620; 8484]	7282 [5540; 10,722]	0.638
Hu IL-1b	1.8 [1.5; 2.2]	2.3 [1.7; 5]	**0.025**
Hu IL-1ra	148.8 [126.2; 194.1]	174.6 [119.8; 211.1]	0.508
Hu IL-4	18.7 [16.9; 21.2]	19.6 [18.2; 21.8]	0.344
Hu IL-5	1.9 [1.1; 2.7]	1.9 [1.4; 2.6]	0.876
Hu IL-6	13.3 [10.1; 24]	16.5 [10.8; 44.1]	0.114
Hu IL-7	7.4 [6; 9.2]	9.2 [7.7; 11.1]	**0.009**
Hu IL-8	43.2 [26.1; 90]	80.1 [35.8; 159.8]	**0.046**
Hu IL-9	33.9 [27.4; 48.5]	34.5 [26.5; 44.4]	0.719
Hu IL-10	19.8 [12.7; 30.1]	24 [16.6; 34.7]	0.269
Hu IL-12 (P70)	104.8 [62.5; 120.2]	124.2 [89.6; 184.7]	**0.018**
Hu IL-13	11.6 [8.6; 14.6]	12.4 [9.6; 15.8]	0.364
Hu IL-17	163.7 [120.4; 182.8]	161.4 [134.4; 220.5]	0.285
Hu Eotaxin	208 [160.9; 263.8]	265.8 [184.3; 317.5]	**0.031**
Hu FGF basic	32.1 [23.3; 36]	36.2 [27.7; 43.3]	0.120
Hu G-CSF	71.7 [60.1; 80.3]	77.4 [65.9; 96.4]	0.154
Hu IFN-g	99.1 [75.7; 118.1]	99.1 [73.2; 127.3]	0.791
Hu IP-10	972 [789; 1317]	1112 [819; 1585]	0.235
Hu MCP-1 (MCAF)	110.6 [72.9; 158.9]	93.4 [62; 179.6]	0.429
Hu MIP-1a	5.4 [4.3; 10]	5.8 [4.1; 10.2]	0.870
Hu MIP-1b	158 [110; 210.5]	149.3 [101.4; 303.2]	0.827
Hu RANTES	30,480 [22,736; 35,399]	31,453 [22,328; 37,531]	0.576
Hu TNF-α	43.2 [31.1; 64]	46.5 [33.6; 68.2]	0.335
Hu VEGF	174 [99.2; 353.9]	298.3 [203.6; 467.8]	**0.012**

In bold type *p*-value < 0.05. HU PDGF-BB—Human platelet-derived growth factor-homodimers B; HU IL-1B—Human interleukin-1 beta; HU IL-1RA—Human interleukin-1 receptor antagonist; HU IL-4—Human interleukin-4; HU IL-5—Human interleukin-5; HU IL-6—Human interleukin-6; HU IL-7—Human interleukin-7; HU IL-8—Human interleukin-8; HU IL-9—Human interleukin-9; HU IL-10—Human interleukin-10; HU IL-12 (P70)—Human interleukin-12 heterodimers 70 kDa; HU IL-13—Human interleukin-13; HU IL-17—Human interleukin-17; HU EOTAXIN—Human Eotaxin; HU FGF BASIC—Human basic fibroblast growth factor; HU G-CSF—Human granulocyte colony-stimulating factor; HU IFN-G—Human interferon gamma; HU IP-10—Interferon gamma-induced protein 10; HU MCP-1 (MCAF)—Monocyte chemoattractant protein-1/monocyte chemotactic and activating factor; HU MIP-1A—Macrophage inflammatory protein-1 alpha; HU MIP-1B—Macrophage inflammatory protein-1 beta; HU RANTES—Human RANTES protein; HU TNF-A—Human tumor necrosis factor alpha; HU VEGF—Human vascular endothelial growth factor.

**Table 3:** Patients’ clinical and biochemical characteristics.

Variable	Non-Calcified	Calcified
AGE (YEARS)	53.6 ± 12.2	67.8 ± 8.7
SEX (M; %)	66	85
DIABETES (%)	8	25
HYPERTENSION (%)	87	100
AGATSTON SCORE (AU)	0	248 (2–734)
BMI (KG/M2)	27.8 ± 5.9	28.9 ± 5.4
E-GFR (ML/MIN)	75 ± 41	71.7 ± 25
DURATION CKD (MONTHS)	24.5 ± 43	17 ± 25
PROTEINURIA (MG/24H)	303 ± 577	255 ± 476
CALCEMIA (MG/DL)	9.5 ± 1.3	9.6 ± 0.5
PHOSPHOREMIA (MG/DL)	3.7 ± 1.4	3.7 ± 0.4
PHOSPHATURIA (MG/24H)	858 ± 370	807 ± 290
PTH (PG/ML)	58 ± 20	53 ± 35
HOMOCYSTEINE (µMOLI/L)	19 ± 9	19 ± 4
CRP (MG/L)	0.4 ± 0.2	0.3 ± 0.1
URIC ACID (MG/DL)	6.0 ± 1.3	6.2 ± 1.1
HEMOGLOBIN (G/DL)	14.2 ± 1.1	14.9 ± 1.9
CHOLESTEROL (MG/DL)	183 ± 48	195 ± 39
HDL-CHOLESTEROL (MG/DL)	51 ± 14	40 ± 11
TRIGLYCERIDES (MG/DL)	130 ± 52	182 ± 80
LDL-CHOLESTEROL (MG/DL)	103 ± 41	110 ± 45
FIBRINOGEN (MG/DL)	315 ± 79	366 ± 78
SAP (MMHG)	127 ± 13	138 ± 8
DAP (MMHG)	80 ± 13	82 ± 5

Legends: BMI—Body mass index; E-GFR—Estimated-glomerular filtration rate; CKD—Chronic kidney disease; PTH—parathyroid hormone; CRP—C-reactive protein; HDL—High-density lipoprotein; LDL—Low-density lipoprotein; SAP—Systolic arterial pressure; DAP—Diastolic arterial pressure.

## Data Availability

Not applicable.

## Supplementary Materials

The following are available online at https://www.mdpi.com/article/10.3390/ijms22136875/s1, Table S1. Correlation between lanthionine and cytokines. Table S2. Characteristics of patients in whom lanthionine was measured
