# Supplementary material for: Lanthionine, a Novel Uremic Toxin, in the Vascular Calcification of Chronic Kidney Disease: The Role of Proinflammatory Cytokines
Source: Int J Mol Sci. 2021 Jun 26;22(13):6875. doi: 10.3390/ijms22136875 (PMC8269354; doi:10.3390/ijms22136875)
Supplement: Supplementary file 1 [file ijms-22-06875-s001.zip › ijms-1219053-supplementary.pdf]

Table S1. Correlation between lanthionine and cytokines.

VARIABLE	CORRELATION WITH LANTHIONINE
**HU PDGF-BB**	−0.154 (p = 0.434)
**HU IL-1B**	0.206 (p = 0.313)
**HU IL-1RA**	0.01 (p = 0.961)
**HU IL-4**	−0.259 (p = 0.183)
**HU IL-5**	−0.038 (p = 0.853)
**HU IL-6**	0.106 (p = 0.59)
**HU IL-7**	−0.183 (p = 0.352)
**HU IL-8**	−0.051 (p = 0.795)
**HU IL-9**	−0.243 (p = 0.214)
**HU IL-10**	−0.176 (p = 0.371)
**HU IL-12(P70)**	−0.082 (p = 0.678)
**HU IL-13**	−0.051 (p = 0.797)
**HU IL-17**	−0.192 (p = 0.337)
**HU EOTAXIN**	−0.245 (p = 0.208)
**HU FGF BASIC**	−0.255 (p = 0.191)
**HU G-CSF**	−0.125 (p = 0.526)
**HU IFN-G**	−0.171 (p = 0.385)
**HU IP-10**	−0.219 (p = 0.264)
**HU MCP-1(MCAF)**	−0.156 (p = 0.429)
**HU MIP-1A**	0.027 (p = 0.89)
**HU MIP-1B**	−0.051 (p = 0.797)
**HU RANTES**	−0.025 (p = 0.901)
**HU TNF-A**	−0.165 (p = 0.402)
**HU VEGF**	−0.046 (p = 0.815)
**DATA ARE EXPRESSED AS SPEARMAN CORRELATION COEFFICIENT (*P*-VALUE).**	

HU PDGF-BB—Human platelet-derived growth factor-homodimers B; HU IL-1B—Human interleukin-1 beta; HU IL-1RA—Human interleukin-1 receptor antagonist; HU IL-4—Human interleukin-4; HU IL-5—Human interleukin-5; HU IL-6—Human interleukin-6; HU IL-7—Human interleukin-7; HU IL-8—Human interleukin-8; HU IL-9—Human interleukin-9; HU IL-10—Human interleukin-10; HU IL-12 (P70)—Human Interleukin-12 heterodimers 70 kDa; HU IL-13—Human interleukin-13; HU IL-17—Human interleukin-17; HU EOTAXIN—Human Eotaxin; HU FGF BASIC—Human basic fibroblast growth factor; HU G-CSF—Human granulocyte colony-stimulating factor; HU IFN-G—Human interferon gamma; HU IP-10—Interferon gamma-induced protein 10; HU MCP-1(MCAF)—Monocyte chemoattractant protein-1/monocyte chemotactic and activating factor; HU MIP-1A—Macrophage inflammatory protein-1 alpha; HU MIP-1B—Macrophage inflammatory protein-1 beta; HU RANTES—Human RANTES protein; HU TNF-A—Human tumor necrosis factor alpha; HU VEGF—Human vascular endothelial growth factor.

Table S2. Characteristics of patients in whom lanthionine was measured.

Patient	Sex	Age	Lanthionine (μM)	GFR (ml/min/1.73 m^2)	Agatston Score
1	F	35	ND	190	0
2	M	45	ND	175	1
3	M	67	1.076	168	2
4	M	62	0.614	140	0

5	F	69	ND	133	0
6	M	56	ND	128	0
7	M	68	ND	122	3
8	F	47	1.130	113	0
9	F	53	ND	112	0
10	M	73	ND	110	3
11	M	50	ND	109	0
12	M	66	0.681	106	1
13	M	59	0.742	99	0
14	M	79	ND	95	1
15	M	68	ND	92	3
16	M	73	ND	91	3
17	F	67	1.021	88	0
18	F	68	ND	88	0
19	M	76	0.640	88	1
20	F	62	ND	85	2
21	F	55	0.970	83	2
22	M	52	ND	82	0
23	M	56	0.938	81	0
24	M	78	ND	79	1
25	F	67	ND	78	0
26	M	69	ND	74	0
27	M	58	0.829	71	0
28	M	75	ND	71	0
29	M	34	ND	71	0
30	M	75	ND	69	3
31	M	66	0.938	67	2
32	M	64	ND	66	2
33	M	36	ND	66	0
34	M	55	0.736	66	0
35	M	49	ND	66	0
36	F	66	ND	63	0
37	F	62	ND	61	0
38	F	79	ND	59	1
39	M	43	ND	54	0
40	M	29	ND	53	0
41	M	80	ND	52	2
42	M	66	ND	49	1
43	M	70	ND	49	0
44	M	53	ND	49	0
45	M	79	0.938	46	3
46	M	56	ND	44	1
47	F	13	ND	43	1
48	M	67	1.316	41	3
49	F	64	ND	40	0
50	M	51	ND	38	0
51	M	54	ND	37	0
52	M	58	ND	36	2
53	M	78	1.076	35	3

54	M	76	1.072	35	2
55	M	43	ND	35	0
56	M	13	ND	34	1
57	M	75	ND	33	1
58	M	72	0.960	32	3
59	M	67	1.255	32	3
60	M	61	ND	32	3
61	M	71	ND	31	3
62	M	43	ND	29	0
63	F	66	0.778	29	1
64	M	64	0.925	28	1
65	M	74	ND	25	1
66	F	67	ND	22	1
67	M	71	0.967	21	2
68	F	71	ND	21	3
69	M	54	0.762	19	1
70	M	37	ND	18	0
71	M	52	ND	14	0
72	M	81	1.447	13	3
73	M	43	1.528	13	1
74	M	45	0.707	11	0

ND: Not detected. Serum samples from all 74 CDK patients were analyzed for lanthionine. In 49 samples, lanthionine was below the LOQ (limit of quantification 0.2 μM in serum matrices-ND). Patient characteristics: Sex, age, lanthionine (μM), Glomerular Filtration Rate (GFR ml/min/1.73 m^2) and the Agatston Score based on patients' TCS (No Agatston score: 0; Agatston score 1: 1–100; Agatston score 2: 101–300; Agatston score 3 > 301).
